# Innovative Utilization of Watermelon By‐Product Powders to Enhance Fresh Pasta Quality: Improving Nutritional Value and Functional Properties

**DOI:** 10.1002/fsn3.70572

**Published:** 2025-07-04

**Authors:** Sultan Acun, Hülya Gül, Fadime Seyrekoğlu

**Affiliations:** ^1^ Department of Food Processing, Suluova Vocational School Amasya University Suluova, Amasya Türkiye; ^2^ Department of Food Engineering, Faculty of Engineering and Natural Sciences Süleyman Demirel University Isparta Türkiye

**Keywords:** antioxidant activity, cooking quality, dietary fiber, pasta enrichment

## Abstract

This study aimed to develop fresh pasta (FP) with improved functional properties by incorporating watermelon by‐product powders (WBP), including watermelon peel powder (WPP), watermelon rind powder (WRP), and watermelon seed powder (WSP). FP samples were formulated with varying substitution levels (0%–15%) using durum wheat semolina, water, and egg. Physicochemical analyses revealed that WPP and WRP above 10% increased moisture content, while WSP reduced it. WBP significantly enhanced ash, fat, and dietary fiber contents, with WSP contributing more to fat and WPP to fiber. Structural changes occurred during cooking; FP with WSP and WRP showed increased thickness, width, and length, while high WPP levels led to structural deterioration. Cooking time increased with fat content, with the longest time recorded for 15% WRP‐FP. Color parameters changed after cooking depending on the formulation. While a values generally decreased, L and b* values exhibited both increases and decreases based on the type and level of watermelon by‐products used. WBP addition improved total phenolic content and antioxidant activity, with the highest phenolic content in 15% WPP‐FP. Texture analysis showed that WBP affected FP firmness, with the highest firmness in 15% WSP‐FP. Sensory evaluation determined optimal WBP levels as 5% WPP, 3.5% WRP, and 10% WSP. These findings highlight WBP's potential for sustainable, nutritionally enhanced FP production.

## Graphical abstract

This study investigates the incorporation of watermelon by‐product powders (peel, rind, and seed) into fresh pasta to enhance its functional properties. The results indicate that these by‐products significantly improve the nutritional composition, antioxidant activity, and texture of the pasta, offering a sustainable approach to food waste utilization. This research underscores the potential of watermelon by‐products as valuable, functional ingredients in the fortification of pasta, contributing to both enhanced product quality and sustainability.

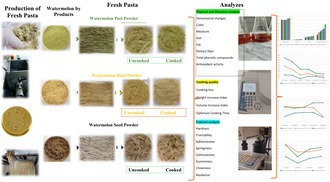

## 1 Introduction

Watermelon ( *Citrullus lanatus* ), a member of the *Cucurbitaceae* family, which also includes melons and squashes, is an annual fruit species. In 2023, approximately 3.2 million tons of watermelon were produced in Turkey, while globally, around 105 million tons of watermelon were produced in 2023 (FAO 2025a). In Turkey, regions such as Manavgat county in Antalya contribute significantly to national watermelon production, accounting for 2.81% and employing different cultivation systems (open field, low tunnel, high tunnel) that influence production costs and profitability (Gül et al. 2023). Watermelon consists of three main components: seeds (2%), rind (30%), and flesh (68%) (Toupal 2022; Ahamad et al. 2022). While the flesh, which is commonly consumed, is the popular part, the rind and peel are generally discarded or used as animal feed in many countries. In some countries, however, watermelon rind is used in the production of jams, pickles, or juices to suppress its undesirable taste (Feizy et al. 2020).

In recent years, due to its rich nutritional value, the use of watermelon rind has increased for the purpose of food enrichment. The rind has been incorporated into various food products, such as cakes and cookies, to enhance their dietary fiber content (Adegunwa et al. 2019). Watermelon rind is rich in dietary fiber, vitamin C, and potassium (Ahamad et al. 2022). It also contains L‐citrulline, a compound that regulates blood pressure and has anti‐aging effects (Mohan and Shanmugam 2016; Ahamad et al. 2022). Additionally, watermelon rind is abundant in phenolic compounds, which contribute to its antimicrobial properties and therapeutic benefits (Neglo et al. 2021) such as antioxidant, anti‐diabetic, antihypertensive, and hepato‐renal protective effects (Kataria and Kaur 2023). Each of these claims is backed by specific studies. For instance, the antihypertensive effect is supported by research indicating that watermelon rind extract can inhibit angiotensin I‐converting enzymes, which are crucial in regulating blood pressure (Romdhane et al. 2017). Sorour et al. (2019) reported that watermelon rind ingestion significantly reduced blood glucose levels in diabetic female albino rats, bringing them closer to normal values, an effect consistent with that of standard anti‐diabetic agents and demonstrating its potential as a natural treatment for hyperglycemia. The treatment with watermelon rind also helped protect against the characteristic weight loss associated with diabetes, as the treated rats showed an insignificant increase in body weight compared to the untreated group. Watermelon rind is rich in natural antioxidants and non‐essential citrulline, as well as minerals such as calcium, magnesium, potassium, and iron (Ho and Che Dahri 2016; Feizy et al. 2020). The peel has analgesic properties, while the rind shows vasodilatory effects (Neglo et al. 2021).

Watermelon seeds also provide significant nutritional benefits. Watermelon seeds contain 25%–30% protein and are rich in peptides with antioxidant properties (Wen et al. 2020). Watermelon seeds contain 57.1% fat, 71.9% of which is unsaturated fat, with 57.4% of this being polyunsaturated fatty acids. They are also rich in essential amino acids (tryptophan, methionine, arginine), minerals such as zinc, iron, phosphorus, and vitamins B1 and B2. These nutrients contribute to the prevention of coronary heart disease and the reduction of blood cholesterol levels (Shahein et al. 2022). The current evidence base for the health effects of watermelon seeds and their powder highlights their potential in promoting vascular health, reducing cancer risk, and providing hepatoprotective benefits. Watermelon seeds are rich in L‐citrulline and L‐arginine, which are crucial for nitric oxide synthesis, thereby improving vascular function and potentially lowering blood pressure (Volino‐Souza et al. 2022). Similarly, Sajjad et al. (2020) found that giving hypertensive adults watermelon seeds daily significantly lowered their blood pressure. This positive effect is likely due to the seeds' high content of beneficial phytochemicals and minerals (cations), which are thought to help relax blood vessels, act as antioxidants, and inhibit an enzyme involved in raising blood pressure. Additionally, studies indicate that watermelon powder supplementation may reduce precancerous lesions in colon cancer models by enhancing nitric oxide production and modulating DNA repair mechanisms (Glenn et al. 2017). Furthermore, watermelon seed extracts have demonstrated protective effects against oxidative liver damage in animal studies (Bazabang et al. 2018).

Given the growing emphasis on sustainable food production and the valorization of agricultural by‐products, the incorporation of watermelon‐derived components into staple foods represents a valuable opportunity. Recent studies have explored the potential of fruit and vegetable residues, including watermelon rind and seed, in baked goods and snacks due to their high fiber, mineral, and antioxidant content (Ashoka et al. 2021; Adegunwa et al. 2019; Zia et al. 2021). However, their application in pasta—especially fresh pasta—remains underexplored. Considering the increasing demand for functional and clean‐label products, integrating such underutilized by‐products into pasta formulations not only supports waste minimization but also contributes to the development of nutritionally enhanced foods with improved functional properties.

Pasta is widely consumed globally as a staple food due to its low cost, long shelf life, and ease of preparation (Lu et al. 2016; Desai et al. 2019). Especially, pasta made from durum wheat offers a nutritious option with its high complex carbohydrate content (74%–77%) and protein content (11%–15%) (Dziki 2021). In 2022, global pasta production reached 16.9 million tons, with Turkey being one of the leading producers, producing approximately 2 million tons in 2023 (TMSD [Turkish Pasta Industrialists Association] 2024). Pasta consumption has increased over the years, with Italy leading the world with a per capita consumption of 23.2 kg, while Turkey's consumption was recorded at 7.6 kg per capita (UNAFPA 2025).

Pasta is produced in two main forms: dry and fresh (Tazrart et al. 2016). Dry pasta is widely preferred due to its long shelf life, while fresh pasta stands out for its flavor, texture, and shorter cooking time. Traditionally, fresh pasta in Italy is made using durum semolina, eggs, and water, and it is consumed in both stuffed and non‐stuffed forms. In Asia, noodles and dumplings are produced using different types of flour. Although the current Pasta Regulation in Turkey defines pasta as a product made solely from *Triticum durum* wheat, fresh pasta is increasingly entering the market in response to growing consumer demand (Madenci 2017).

From a nutritional perspective, traditional pasta, despite its high carbohydrate content, is low in dietary fiber (0.9–1.9 g/100 g) and phenolic compounds (Romano et al. 2021; Wang et al. 2022; Long et al. 2024). According to reports by the World Health Organization (WHO) and the Food and Agriculture Organization (FAO), it is recommended to consume at least 25 g of dietary fiber daily, emphasizing the need to increase the intake of fruits, vegetables, and whole‐grain products (FAO 2025b). Sufficient dietary fiber intake is known to be associated with a reduced risk of digestive system disorders, diabetes, colon cancer, and cardiovascular diseases (Acun and Gül 2014; Mai et al. 2022). Moreover, pasta is also deficient in antioxidants, which play a protective role against aging and various chronic diseases (Neglo et al. 2021; Long et al. 2024).

In recent years, research aimed at enhancing the nutritional value of pasta has focused on the production of pasta enriched with functional components. In this regard, whole grain flours (wheat, oats, barley, rice, etc.) are commonly used in pasta production, and plant powders obtained from fruits, vegetables, herb seeds, and edible flowers are incorporated into formulations. Additionally, methods such as replacing water with fruit juice or vegetable purees in dough preparation to increase dietary fiber and antioxidant content have gained attention. Agricultural and food industry by‐products, particularly fruit waste, are being used as functional ingredients in pasta production (Long et al. 2024). Studies have shown that adding components such as mushroom powder (Lu et al. 2016), almond flour (Martínez et al. 2017), fava bean powder (Tazrart et al. 2019), and β‐glucan (De Santis et al. 2020) to fresh pasta can increase its nutritional value. However, the addition of these ingredients requires the development of proper formulations to ensure that the sensory quality of pasta is not negatively impacted.

In conclusion, when enriched with functional components, fresh pasta has the potential to offer both a nutritious and healthy food alternative. However, appropriate production processes must be developed to ensure that nutritional improvements do not adversely affect sensory and technological quality. In this context, the production of functional pasta enriched with fiber and antioxidants, with high consumer acceptability, remains an important area of research and food industry development in the future.

Watermelon rind and seeds have been used in the enrichment of cereal products such as pasta and cakes. However, no studies on the use of peel, rind, or seeds in fresh pasta production, which has gained popularity in recent years, have been found in the literature. Particularly, research on the biological effects of the peel and its application in food products is limited. It is crucial to conduct studies aimed at increasing the use and awareness of nutrient‐rich peel and seed in food products. This study aims to determine the effects of using WPP, WRP, and WSP in the production of fresh pasta on its nutritional composition, total phenolic content, antioxidant activity, cooking quality, texture, and sensory characteristics.

## 2 Materials and Methods

### 2.1 Material

**FIGURE 1:**
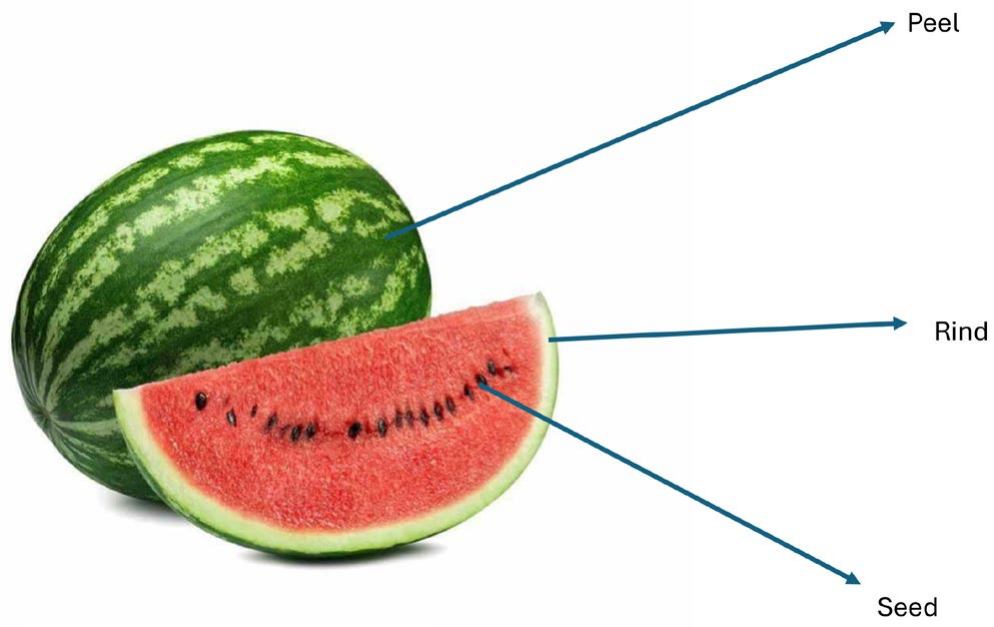
Watermelon components: Watermelon peel, rind, and seed.

The *Crimson Sweet* variety of watermelon was purchased from a local producer in June 2022. WPP, WRP, and WSP were prepared by following the methodology detailed in our previous study (Acun et al. 2025). Briefly, the watermelons were promptly transported to the Food Analysis Laboratory at Amasya University Suluova ocational School, where the external surfaces were carefully washed. The rind and peel were then meticulously separated, with the rind being cut into 8‐mm thick pieces. The seeds were extracted from the inner portion of the watermelon and washed with tap water to remove any residual watermelon flesh and sugar. The watermelon rinds, peels, and seeds (Figure 1) were dried in an oven (Nuve, FN300, Turkey) at 50°C–55°C for 12–14 h. The dried samples were ground using an industrial‐type grinder (Arsel, ARSO3, Konya/Turkey). The ground samples were passed through sieves with a mesh size of 325 μm and stored in airtight packages for use in pasta production.

Semolina from durum wheat was obtained from Selva Pasta Factory (Konya, Turkey), and fresh whole eggs were purchased from a local market in Isparta, Turkey. Analytical‐grade chemicals were sourced from Merck, including Folin–Ciocalteu reagent (for total phenolic content analysis), 2,2‐diphenyl‐1‐picrylhydrazyl—DPPH (for antioxidant activity assay), and gallic acid (as a standard for calibration). A dietary fiber analysis kit was obtained from Megazyme.

### 2.2 Fresh Pasta Production

The production method was adapted from those described by Carini et al. (2009) and Lemes et al. (2012). The process involved using semolina with moisture (12.38%), ash (0.51%), and total dietary fiber content (6.89%), along with water, fresh whole egg, and watermelon by‐products. The pasta dough was prepared by kneading for 10 min using a La Monferrina Dolly pasta machine (Italy). To determine the optimal formulation, trials were conducted with varying water and egg proportions. Based on preliminary trials, the egg quantity was fixed at 55 g, and a fixed amount of water—30 g per 100 g of semolina—was added to all formulations. This amount was determined to maintain a target dough moisture level between 30% and 33%, and was kept constant to evaluate the influence of watermelon by‐products independently. Moisture content was monitored gravimetrically during preliminary tests to ensure consistency. This approach was intended to ensure that any differences observed in the final moisture content would result solely from the intrinsic water‐holding capacities of the incorporated watermelon by‐products. The doughs were then shaped using a 6 mm‐wide tagliatelle cutting die compatible with the La Monferrina Dolly pasta machine, forming long flat ribbons.

The watermelon by‐products were ground and passed through a 325‐μm sieve before incorporation into the dough. These by‐products were added at a maximum substitution rate of 15% on a weight basis. The addition levels were as follows: for watermelon peel, 0%, 2.5%, 5%, 7.5%, 10%, and 15%; for watermelon rind, 3.75%, 7.5%, 11.25%, and 15%; and for watermelon seeds, 5%, 10%, and 15%. Although watermelon peel was tested up to a 15% substitution, significant structural deterioration in the dough was already observed at this level due to its very fine particle structure. In preliminary trials, higher levels were tested but were not acceptable in terms of sensory quality; thus, 15% was determined as the upper acceptable limit. Watermelon rind ensured that the dough was whole and elastic due to the sugar and protein in its structure. In addition, since it had a high appreciation score in sensory terms in preliminary tests, the addition rate was determined to be higher. Watermelon seed improved the dough structure due to its high oil content, but due to its structure, the particles obtained during grinding negatively affected the sensory quality. Therefore, different ratios were preferred in adding watermelon by‐products to fresh pasta. These proportions were optimized based on the technological and sensory properties observed during preliminary trials.

### 2.3 Determination of Chemical and Physical Properties

The analysis of moisture content, ash, fat, and total dietary fiber in fresh pasta was performed according to AACC methods 44‐01.01, 08‐01.01, 30‐25.01, and 32‐07.01. (AACC 2000). Moisture analyses were conducted immediately after pasta production, without any storage or delay.

Pasta color was determined by a reflectance colorimeter (Minolta Chroma meter CR‐410, Japan) following the CIE–L* a* b* color system. Raw and optimally cooked pasta brightness (L*) and color (+a: red −a: green; +b: yellow; −b: blue) were evaluated. Before measurement, cooked pasta was carefully dried with absorbent paper (Simonato et al. 2021). To assess perceptible color changes caused by cooking, total color difference (ΔE) was calculated between raw and cooked pasta using the following equation:

$$\mathrm{\Delta E}=\sqrt{\left[{\left(L_{2}*-L_{1}*\right)}^{2}+{\left(a_{2}*-a_{1}*\right)}^{2}+{\left(b_{2}*-b_{1}*\right)}^{2}\right]}$$

where *L*_1_, *a*_1_, and *b*_1_ refer to the color values of uncooked pasta and *L*_2_, *a*_2_, and *b*_2_ to those of cooked pasta. This calculation provides an objective measure of the overall color change between the two states.

Fresh pasta was cut into an average length of 4 cm after production. Width, thickness, and length measurements were made from three different points of the cut pasta with a digital caliper. Similarly, these measurements were taken from cooked pasta, and the dimensional changes of the pasta were expressed as a percentage (Lucisano et al. 2012).

### 2.4 Cooking Quality

The optimum cooking time (OCT) of fresh pasta was determined according to AACC method 66‐50.01 (AACC 2000). Cooking loss (CL) was evaluated using the gravimetric procedure outlined by Elgün et al. (2002). Water absorption and volume expansion ratios were calculated based on weight and volume changes after cooking at the optimal duration, following the method described by Kaur et al. (2012).

### 2.5 Determination of Total Phenolic Compound and Antioxidant Activity

To evaluate the total phenolic content (TPC), phenolic compounds were extracted from raw materials and fresh pasta samples. For fresh pasta, 1 g of the sample was combined with 10 mL of a solvent mixture (ethanol: acetic acid: water, 50:8:42) and homogenized using an Ultraturrax at 25,000 rpm for 3 min. The resulting mixture was centrifuged (Hermle Z206A, Germany) at 4427 ***g*** for 30 min and filtered, and the filtrate was used for TPC analysis. The TPC of watermelon by‐products was measured following the Singleton and Rossi (1965) method, whereas the TPC of fresh pasta was determined using the method described by Marinelli et al. (2015). Results were reported as mg gallic acid equivalent (GAE) per gram of sample.

Antioxidant activity of raw materials and fresh pasta was assessed using the DPPH radical scavenging assay, based on the method of Aksoylu (2012). For this analysis, 200 μL of extract was mixed with 3.8 mL of DPPH solution and incubated in the dark for 1 h. The absorbance was then recorded at 515 nm, and antioxidant activity was expressed as DPPH radical scavenging percentage (% inhibition), as commonly used in DPPH‐based antioxidant assays.

### 2.6 Texture Profile Analysis (TPA)

The textural properties of pasta were assessed using a Texture Analyzer (TA‐XT plus, Stable Micro Systems Ltd., UK) with a modified method based on Padalino et al. (2013) and Larrosa et al. (2016). Three pasta strands, cooked to optimum cooking time, were aligned side‐by‐side on the base plate and compressed twice using a 2 cm diameter flat cylindrical probe to generate compression‐relaxation stress profiles. The compression distance was set to 50% of the original pasta thickness. From the time‐force curve, firmness, cohesiveness, chewiness, and resilience were determined (Szczesniak 2002). Test parameters were configured as follows: pre‐test speed of 1 mm/s, test speed of 5 mm/s, post‐test speed of 5 mm/s, and a 5 kg load cell.

### 2.7 Sensory Evaluation

The sensory evaluation of OCT pasta samples was conducted with a panel of 11 informed judges. The panel identified 11 sensory attributes, including color, odor, appearance, stickiness, taste, firmness, clumping, texture, overall acceptability, and affordability.

For the evaluation, 200 g of each pasta sample was cooked at the optimum cooking time (OCT) in 1 L of distilled water, and 15 g of cooked pasta was served to each judge in a covered dish immediately after cooking. The presentation order of the samples was balanced and randomized among the judges. A seven‐point scale was employed for all sensory attributes, where 1 indicated the lowest intensity and 7 the highest. The average scores for each attribute were calculated.

In addition, judges rated the overall acceptability of each sample using a verbal seven‐point hedonic scale, where 1 represented “dislike extremely” and 7 represented “like extremely.” Pasta samples were considered acceptable if their mean overall acceptability score was 5 or higher, corresponding to “like slightly” on the 7‐point hedonic scale (Simonato et al. 2021).

Affordability, reflecting the panelists' perception of price–performance balance, was evaluated separately using a five‐point scale (1 = very low affordability, 5 = very high affordability).

### 2.8 Statistical Analysis

All analyses were performed in triplicate or more, and results were expressed as mean ± standard deviation. Prior to conducting the analysis of variance (ANOVA), the assumptions of normality and homogeneity of variances were tested using the Shapiro–Wilk and Levene's tests, respectively. Differences among means were evaluated using one‐way ANOVA, followed by Duncan's post hoc test at a significance level of *p* < 0.05. Statistical analyses were performed using SPSS software (version 24.0; SPSS Inc., Chicago, IL, USA).

## 3 Results and Discussion

### 3.1 Determination of Physical and Chemical Properties

The mean values of the moisture, ash, total fat, and total dietary fiber contents of FP produced by substituting durum wheat semolina with WPP, WRP, and WSP at different ratios are given in Table 1. Substituting WPP and WRP at low ratios (up to 7.5%) resulted in moisture contents identical to the control. However, as the ratio increased, the moisture content of the FP also rose. This is likely due to an increase in the dietary fiber content of FP, which occurs when WPP and WRP are incorporated in increasing ratios. It is well known that dietary fibers can absorb water about ten times their weight.

**TABLE 1:** Chemical composition of fresh pasta enhanced with watermelon by‐product powders (WPP, WRP and WSP).

Substitution levels of WPP, WRP and WSP (%)	Moisture (%)	Ash (%)	Fat (%)	Total dietary fiber (%)
0 (Control)	30.64 ± 0.20^b^	0.63 ± 0.02^g^	3.92 ± 0.44^i^	8.10 ± 0.30^j^
WPP				
2.5	30.27 ± 1.45^b^	0.85 ± 0.14^f^	4.83 ± 0.59^ef^	11.84 ± 0.49^h^
5	30.43 ± 0.26^b^	0.97 ± 0.15^ef^	5.09 ± 0.36f^gh^	13.28 ± 0.05^f^
7.5	30.78 ± 0.39^b^	1.20 ± 0.06^cd^	5.26 ± 0.38^efg^	15.44 ± 0.40^d^
10	32.44 ± 0.18^a^	1.27 ± 0.21^cd^	5.66 ± 0.58^efgh^	17.18 ± 0.49^b^
15	33.31 ± 0.31^a^	1.41 ± 0.04^bc^	6.53 ± 0.74^d^	23.78 ± 0.07^a^
WRP				
3.75	30.37 ± 0.48^b^	1.11 ± 0.09^de^	4.77 ± 0.10^gh^	12.44 ± 0.17^gh^
7.5	30.43 ± 2.91^b^	1.33 ± 0.24^bc^	4.27 ± 0.19^hi^	14.62 ± 0.87^e^
11.25	32.52 ± 0.34^a^	1.53 ± 0.05^b^	4.86 ± 0.33^fgh^	15.82 ± 0.11^cd^
15	33.98 ± 0.30^a^	1.79 ± 0.09^a^	5.89 ± 0.75^de^	16.40 ± 0.04^c^
WSP				
5	28.23 ± 0.17^c^	1.11 ± 0.07^de^	8.70 ± 0.15^c^	10.54 ± 0.19^i^
10	27.95 ± 0.15^c^	1.21 ± 0.06^cd^	9.54 ± 0.22^b^	12.92 ± 0.20^fg^
15	27.38 ± 0.60^c^	1.28 ± 0.04^cd^	10.44 ± 0.38^a^	15.91 ± 0.76^cd^

*Note:* ^a–i^There is no statistical difference between the data shown with the same letter in the same column (one‐way ANOVA; Duncan test; *p* ≤ 0.05).

Abbreviations: FP, Fresh pasta; WPP, Watermelon peel powder; WRP, Watermelon rind powder; WSP, Watermelon seed powder.

However, the addition of WSP resulted in a decrease in the moisture values of the FP compared to the control, other WSP, and WRP‐supplemented FP. It is noteworthy that no statistical difference was observed between the different addition rates of WSP. The reason for the lower moisture content of the FP‐containing WSP can be attributed to their higher fat content. The decrease in moisture resulting from the replacement of fat‐rich WSP can be attributed to the hydrophobic nature of lipid molecules, which are regarded as the most effective barriers to water transfer and exhibit varying interactions with water. Fat molecules create a barrier during pasta production, inhibiting water molecule penetration, which likely contributes to the low moisture content observed in these pastas (Morillon et al. 2002; Bourlieu et al. 2006).

In general, the ash content of fresh pasta increased with the addition of watermelon by‐products compared to the control. The sample with the highest ash content was 15‐WRP‐FP. This is due to the higher ash content of WRP compared to WPP and WSP (Acun et al. 2025). The substitution of WPP above 5% significantly impacted the ash value; at 15% WPP, the ash content reached 1.41%, compared to 0.63% in the control. This suggests that, while minimal addition levels (up to 5%) of WPP may not alter the mineral content of the fresh pasta considerably, a more substantial incorporation leads to a noticeable increase. However, although WSP significantly increased the ash content compared to the control sample, the ash values were not statistically influenced by increasing levels of WSP substitution. Consistent with our findings, Lima et al. (2015) and Çelik and Işık (2023) reported increased ash content in gluten‐free cookies and muffins, respectively, when watermelon rind powder (WRP) was used. According to their analyses, WRP contains significant amounts of minerals such as magnesium (465.36 mg/100 g) and potassium (3381 mg/100 g), which contribute to the overall ash content in the final products. Similarly, Ashoka et al. (2021) found that the control cookies contained lower levels of calcium (11.19 mg), iron (0.80 mg), and phosphorus (50.20 mg) compared to the cookies with 30% WRP incorporation, which had calcium (43.11 mg), iron (2.30 mg), and phosphorus (71.81 mg), respectively. The disparity in mineral composition may be attributed to the higher mineral content in WRF compared to refined wheat flour. The inclusion of WRP not only helps in utilizing waste but also enriches cereal‐based products like pasta with essential minerals, contributing to their nutritional value.

The fat level in fresh pasta varies considerably depending on its ingredients and production techniques. Traditional fresh pasta, predominantly composed of durum wheat semolina, often exhibits minimal fat content; however, variances arise from the use of other ingredients such as eggs or enriched components like WBP in the current study. The fat content of all FP samples containing WBP considerably increased in comparison to the control. The most substantial rise in FP samples occurred in those substituted with WSP, where fat content increased from 3.92% in the control to 10.44% at 15% WSP. Even at low levels, such as 5%, the inclusion of WSP elevated the fat content of FP more significantly than the maximum additions of WPP and WRP (15%). This is attributable to the elevated fat content of WSP relative to WPP and WRP, as established in our previous research (Acun et al. 2025).

Traditional fresh pasta made without eggs generally contains about 1%–2% fat, primarily from the wheat. However, the control FP in this study was prepared with egg, which may account for its higher fat content (3.92% ± 0.44%), as egg yolk contributes significantly to lipid levels (Alamprese et al. 2009). Various studies indicate that watermelon seed oil contains a significant percentage of essential fatty acids, particularly linoleic acid, oleic acid, and palmitic acid (Eke et al. 2021; Chaudhari et al. 2022; Kaymak et al. 2022). Consequently, WSP provides a significant source of polyunsaturated fatty acids alongside other bioactive compounds, making it an appropriate choice for the development of an innovative functional food product. In this regard, it can be suggested that the WBPs are a better alternative to some other contributions to enrich FP. Comparative studies highlight the advantages of WSP over other alternatives, such as chia‐based products. Aja and Haros (2022) found that FP, to which they added whole chia flour, chia fiber, and chia protein, had a lower lipid level than the control sample (100% wheat flour) at a 10% substitution level, which is in contrast to our findings.

The content of dietary fiber in FP exhibited a substantial increase with the incorporation of WBP and with escalating levels of addition. The reason for this was that WBP had a higher dietary fiber content than durum wheat semolina. Comparing WPP, WRP, and WSP revealed that WPP was more effective in enhancing the dietary fiber content of FP. This was due to the higher total dietary fiber content of WPP (78.55%) than WRP (48.66%) and WSP (16.28%) (Acun et al. 2025). Specifically, WPP increased dietary fiber content from 8.10% in the control to 23.78% at 15% WPP, while WRP increased it to 16.40% at the same substitution level. While all three types of WBP markedly increased dietary fiber levels when added at a concentration of 15%, the most significant effect was noted in FP containing WPP at the same concentration. The WBP utilized in our investigation was more successful in enhancing the dietary fiber content of FP than in comparable studies. In this context, the increase in fiber content of pasta from 3.5% to 11.5% was reported by Long et al. (2022) when the WRP ratio in the pasta recipe was changed from 0% to 25%. Compared to the dietary fiber content (11.3 g/100 g) of pasta containing 25% spinach leaf powder (Iacobellis et al. 2024), the dietary fiber content of FP fortified with WRP, WPP, and WSP was higher, despite being used at much lower supplementation levels. In conclusion, the incorporation of WBP into FP significantly enhances its dietary fiber content, with WPP emerging as the most effective agent among the tested variants. The substantial increase in dietary fiber levels, particularly with minimal additions of WPP and WRP, highlights the potential of these by‐products to improve the dietary fiber content of FP. Moreover, dietary fiber is important for health as it reduces the incidence of chronic diseases such as cardiovascular disease, diabetes, and certain cancers; contributes to overall gut health; promotes satiety; and enhances immune function (Bulsiewicz 2023; Hayyat et al. 2023; Yin et al. 2024; Marc et al. 2024). The European Food Safety Authority (EFSA) recommends a daily intake of 25 g of dietary fiber for adults. The enriched pasta samples met this requirement to varying extents, while the 15% WPP‐enriched FP can almost satisfy an adult's daily dietary fiber requirement when consumed at an amount of 100 g/day.

### 3.2 Dimensional Changes of Fresh Pasta Produced by Adding Watermelon By‐Products

Cooking time is a critical factor that influences the extent of dimensional changes in pasta. In this study, the optimal cooking time (OCT) varied depending on the type and level of watermelon by‐product (WBP) added. Specifically, the OCT for pasta enriched with watermelon seed powder (WSP) was longer, which may have contributed to the greater dimensional changes observed at higher substitution levels.

Cooking induces a range of physical and chemical transformations in pasta, primarily involving water absorption, starch gelatinization, and protein matrix softening, which lead to dimensional changes such as increases in thickness and width. While the length of pasta is generally less affected, slight elongation or shrinkage may occur depending on capillary action and the flexibility of the pasta during cooking (Hwang et al. 2022). Furthermore, starch swelling and gluten network degradation contribute to increased surface roughness, resulting in a more irregular texture (Ohmura et al. 2021).

In the present study, as shown in Table 2, dimensional changes varied depending on the type of watermelon by‐product (WBP) used. Pasta enriched with watermelon rind powder (WRP) exhibited the most significant increases in thickness and width, likely due to its higher sugar and protein content, which enhance water retention and swelling during cooking (Acun et al. 2025). Additionally, decreasing WRP particle size has been associated with elevated starch and dietary fiber levels (Long et al. 2024), further promoting water binding and expansion. These characteristics also contributed to the highest volume and weight increases observed in WRP‐containing samples, particularly at higher substitution levels (Table 4).

**TABLE 2:** Dimensional changes in fresh pasta before and after cooking with the addition of watermelon by‐products.

Substitution levels of WPP, WRP and WSP (%)	Length (%)	Thickness (%)	Width (%)
0 (Control)	15.85 ± 5.64^abc^	37.50 ± 0.00^a^	36.00 ± 4.84^ab^
WPP			
2.5	14.27 ± 3.11^bc^	24.21 ± 9.54^abc^	33.66 ± 2.15^b^
5	12.93 ± 4.82^cd^	15.74 ± 8.01^bcd^	25.56 ± 5.77^bc^
7.5	9.60 ± 5.66^cd^	12.50 ± 1.65^cd^	18.61 ± 2.36^cd^
10	9.36 ± 2.98^cd^	4.17 ± 3.25^de^	9.74 ± 2.73^de^
15	4.80 ± 0.49^d^	7.41 ± 6.41^e^	7.60 ± 8.20^e^
WRP			
3.75	22.85 ± 6.11^ab^	35.65 ± 9.85^ab^	33.42 ± 2.81^b^
7.5	24.18 ± 0.32^a^	35.33 ± 8.96^ab^	35.63 ± 3.61^ab^
11.25	24.08 ± 3.22 ^a^	35.12 ± 9.16^ab^	45.01 ± 2.81^a^
15	14.42 ± 2.54 ^bc^	15.74 ± 8.01^bcd^	45.11 ± 7.87^a^
WSP			
5	5.04 ± 1.98^d^	24.07 ± 1.60^abc^	16.01 ± 3.53^cde^
10	13.01 ± 3.32 ^cd^	24.54 ± 3.20^abc^	28.99 ± 0.86^b^
15	16.74 ± 3.74 ^abc^	28.24 ± 8.13^abc^	32.12 ± 4.33^b^

*Note:* ^abc^There is no statistical difference between the data shown with the same letter in the same column (one‐way ANOVA; Duncan test; *p* ≤ 0.05).

Abbreviations: FP, Fresh pasta; WPP, Watermelon peel powder; WRP, Watermelon rind powder; WSP, Watermelon seed powder.

In contrast, pasta produced with watermelon peel powder (WPP) exhibited a decrease in length, thickness, and width as the addition level increased. Among all samples enriched with watermelon by‐products, the lowest proportional dimensional changes were observed in the 15‐WPP‐FP samples. This reduction is likely due to the disruption of the gluten network caused by the fine particles of watermelon peel, which weaken structural integrity during cooking and lead to disintegration.

In fresh pasta enriched with WSP, increases in length, width, and thickness were positively correlated with the addition level. WSP is known to be a rich source of fiber, carbohydrates, and proteins (Gadalkar and Rathod 2020). These components are believed to enhance water retention during cooking, contributing to the observed dimensional expansion compared to the uncooked samples.

Lucisano et al. (2012) reported that dimensional changes in pasta due to cooking can range from 39.19% to 61.52%, depending on formulation and cooking conditions. In our study, the observed changes align with this range and confirm that both the composition and cooking time significantly influence pasta geometry during processing.

Overall, the type and level of watermelon by‐product added to fresh pasta had a considerable impact on dimensional properties. While WRP and WSP promoted expansion during cooking, WPP—especially at higher levels—was associated with reduced dimensional stability, likely due to structural weakening.

### 3.3 Color Properties of Fresh Pasta Produced by Adding Watermelon By‐Products

The color characteristics of fresh pasta produced by adding watermelon by‐products are given in Table 3.

**TABLE 3:** Color properties of fresh pasta produced by adding watermelon by‐products.

Substitution levels of WPP, WRP and WSP (%)	Uncooked			Cooked			ΔE
	L*	a*	b*	L*	a*	b*	
0 (Control)	72.59 ± 2.16^aA^	1.12 ± 0.15^cdA^	20.11 ± 0.19^abA^	70.71 ± 0.70^aA^	−0.95 ± 0.05^dB^	19.58 ± 0.58^aA^	3.05 ± 1.22^cd^
WPP							
2.5	61.08 ± 0.91^dB^	−3.10 ± 0.32^eA^	16.49 ± 0.29^cA^	64.57 ± 0.26^dA^	−4.57 ± 0.14^fB^	16.95 ± 0.17^bcA^	3.85 ± 1.01^cd^
5	60.83 ± 0.48^dA^	−3.42 ± 0.20^eA^	14.05 ± 0.31^dB^	60.44 ± 0.47^fA^	−5.31 ± 0.06^gB^	16.92 ± 0.76^bcA^	3.26 ± 0.54^cd^
7.5	56.72 ± 0.27^eB^	−3.92 ± 0.02^fA^	14.02 ± 0.48^dB^	59.59 ± 0.48^fA^	−5.67 ± 0.22^hB^	16.65 ± 0.04^cdA^	4.47 ± 0.68^cd^
10	52.55 ± 0.39^fB^	−4.09 ± 0.66^fgA^	11.78 ± 0.06^efB^	57.39 ± 0.31^gA^	−6.23 ± 0.08^iB^	15.97 ± 0.29^dA^	4.12 ± 0.74^cd^
15	50.25 ± 0.67^gB^	−4.46 ± 0.14^gA^	11.14 ± 1.20^fB^	55.05 ± 1.02^hA^	−6.94 ± 0.19^jB^	14.39 ± 0.39^eA^	9.12 ± 1.27^a^
WRP							
3.75	66.24 ± 0.64^bB^	0.86 ± 0.14^dA^	20.47 ± 2.42^aA^	68.31 ± 1.01^bA^	−1.21 ± 0.03^eB^	16.88 ± 0.37^bcA^	4.71 ± 2.25^bc^
7.5	64.81 ± 0.38^bB^	1.20 ± 0.11^cdA^	19.70 ± 0.12^abA^	66.54 ± 0.36^cA^	−0.81 ± 0.02^dB^	17.57 ± 0.23^bB^	3.51 ± 0.20^cd^
11.25	61.51 ± 0.51^cdB^	1.35 ± 0.38^cA^	19.10 ± 0.25^abA^	65.57 ± 0.37^cdA^	−0.47 ± 0.08^cB^	17.28 ± 0.51^bcB^	4.84 ± 0.60^bc^
15	61.47 ± 0.17^cdB^	1.37 ± 0.05^cA^	18.77 ± 0.20^bA^	65.70 ± 0.83^cdA^	−0.45 ± 0.10^cB^	16.94 ± 0.77^bcB^	4.99 ± 0.52^bc^
WSP							
5	63.00 ± 0.56^cA^	2.16 ± 0.02^bA^	12.62 ± 0.14^eB^	61.91 ± 0.33^eB^	1.96 ± 0.04^bB^	14.74 ± 0.16^eA^	2.44 ± 0.45^d^
10	57.27 ± 0.58^eA^	2.40 ± 0.08^abB^	10.54 ± 0.18^fB^	54.63 ± 0.99^hiA^	2.72 ± 0.08^aA^	12.36 ± 0.29^fA^	3.37 ± 1.14^cd^
15	47.35 ± 1.93^hB^	2.63 ± 0.50^aA^	8.66 ± 0.49^gB^	53.82 ± 0.74^iA^	2.88 ± 0.06^aA^	10.23 ± 0.31^gA^	6.69 ± 2.03^b^

*Note:* ^a–i^There is no statistical difference between the data shown with the same letter in the same column. ^AB^There is no statistical difference between applications shown with the same letter (one‐way ANOVA; Duncan test; *p* ≤ 0.05).

Abbreviations: FP, Fresh pasta; WPP, Watermelon peel powder; WRP, Watermelon rind powder; WSP, Watermelon seed powder.

**FIGURE 2:**
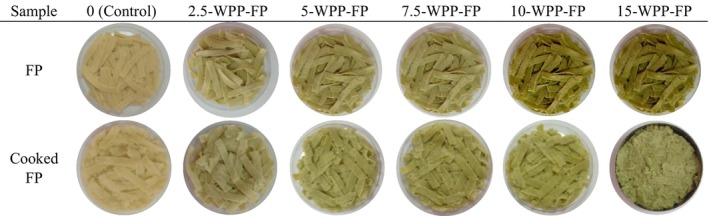
Fresh and cooked pasta supplemented with different levels of (0%, 2.5%, 5%, 7.5%, 10%, 15%) watermelon peel powder, FP, Fresh pasta; WPP, Watermelon peel powder.

**FIGURE 3:**
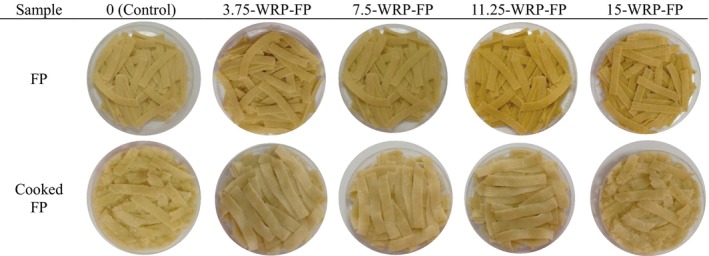
Fresh and cooked pasta supplemented with different levels of (0%–15%) watermelon rind powder, FP, Fresh pasta; WRP, Watermelon rind powder.

**FIGURE 4:**
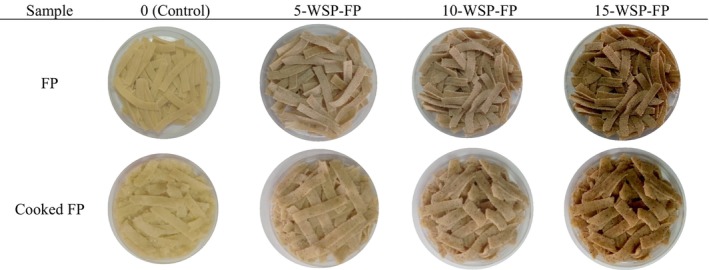
Fresh and cooked pasta supplemented with different levels of (0%–15%) watermelon seed powder. FP, Fresh pasta; WSP, Watermelon seed powder.

Color analysis of the prepared pasta samples was performed before and after cooking. Images of pasta produced by adding WPP are presented in Figure 2, images of WRP in Figure 3, and images of WSP in Figure 4. The highest L* value was found in the control group, while the lowest L* value was found in the 15WSP‐FP. The addition of watermelon by‐products caused a decrease in L* values and caused the pasta to be darker in color. As the addition rate of all watermelon by‐products increased, the L* value decreased and the pasta colors became darker. The highest a* value was found in pasta with 15% watermelon seeds, while the lowest a* value was found in 15‐WRP‐FP. As the amount of watermelon outer rind powder added increased, the green color increased. The increase in the amount of watermelon inner rind powder caused the red color to increase. When the b* values were examined, the highest b* value was observed in 3.75‐WRP‐FP, while the lowest b* value was observed in pasta with 15‐WSP‐FP. As the addition rate of all watermelon by‐product powders increased, a decrease in b* values was observed.

Color values of cooked pasta were examined. Similar to the uncooked pasta, the highest L* value was observed in the control group, and the lowest L* value was observed in 15‐WSP‐FP. The addition of watermelon outer rind, inner rind, and seed powder caused the pasta to be darker in color. The change in the a* value of cooked pasta was similar to that of uncooked pasta. As the added watermelon outer rind powder increased, the green color increased. The increase in the ratio of watermelon inner powder in cooked pasta caused a decrease in the green color. The increase in watermelon seed powder caused an increase in red color. In the b* values, similar to the uncooked pasta, the increase in the ratio of outer shell powder caused a decrease in b* values. On the contrary to the uncooked pasta, the increase in the ratio of watermelon inner rind powder caused an increase in b* values. Similar to uncooked pasta, watermelon seed powder showed a decrease with increasing ratio.

In the study conducted by Long et al. (2022), the use of watermelon rind flour in pasta and its effect on quality and color values were investigated. The use of watermelon by‐products significantly affected the quality parameters. In the study, the darkness of the pasta increased (L* value decreased) and the intensity of yellowness increased (b* value increased), while the change in redness (a* value) was minimal. Pasta containing 10%–20% watermelon rind flour was considered high fiber with acceptable sensory scores despite the color changes.

The other research discusses the utilization of watermelon by‐products, especially seeds, which can be used in food products such as pasta. Furthermore, the use of watermelon by‐products can affect the color values of pasta, contributing to a more attractive visual appearance. This approach is in line with sustainable practices by reducing waste and promoting the use of underutilized food resources (Vinhas et al. 2021). Color values were also investigated in pasta using lycopene‐rich watermelon concentrate as a natural food colorant (Galdeano et al. 2022). In this study, lycopene level caused significant changes in the color of the product. As L* values decrease (darken) a* coordinate the higher the lycopene supplementation levels (the redder) increased in all products. Products with higher a* values have lower L* values, similar to those reported by Dehghan‐Shoar et al. (2010).

Similar results were obtained by Bhat and Ahsan (2015) lycopene‐containing cookies have reduced aperture, while lycopene, as the amount of pigment increased, the redness values also increased. An increase in the a* coordinate with increasing extract content is associated with the reddish color of watermelon concentrate. Da Costa et al. (2010) also reported that the intensity of the red color of extruded watermelon products increased as lycopene levels increased. Zhu et al. (2010) reported changes in color parameters. For noodles with low pigment levels (up to 0.5%), cooking reduced the lightness levels, while at high concentrations (> 1.0%) it showed the opposite effect, which was dose‐dependent in cooked noodles.

In this study, similarly, as the added watermelon by‐products increased, decreases in L* value were observed, while differences were observed in a* value. As the proportion of watermelon rind increased, a* value decreased, and as the proportion of watermelon inner rind and watermelon seeds increased, a* value increased.

The total color difference (ΔE) values of fresh pasta samples varied between 2.44 and 9.12, depending on the type and concentration of the incorporated watermelon by‐product (Table 3). According to the perceptibility thresholds defined by Green (2023), ΔE values between 3 and 6 are considered clearly perceptible, while values above 6 indicate a strongly noticeable difference. In this context, most samples—including the control (3.05 ± 1.22)—exhibited color differences that fall within the clearly perceptible range. The sample containing 15% substitution demonstrated the highest ΔE value (9.12 ± 1.27), reflecting a highly distinguishable color change. Conversely, samples with lower substitution levels (e.g., 2.44 ± 0.45) exhibited minimal to moderate visual differences.

Statistical analysis revealed significant differences among the samples (*p* < 0.05), indicating that both the type and level of watermelon by‐product addition had a substantial impact on the visual attributes of fresh pasta. In general, higher substitution levels were associated with greater total color differences, suggesting a direct relationship between by‐product concentration and perceptible color change. These findings highlight the influence of functional ingredient incorporation on consumer‐relevant quality parameters such as appearance.

### 3.4 Cooking Quality

Cooking quality of pasta is affected by various factors, including the ingredients used, cooking methods, and physical properties of the pasta. Cooking quality of fresh pasta produced by adding watermelon by‐products is given in Table 4.

**TABLE 4:** Cooking quality of fresh pasta produced by adding watermelon by‐products.

Substitution levels of WPP, WRP and WSP (%)	Cooking Loss (%)	Weight Increase Index (%)	Volume Increase Index (%)	OCT (min)
0 (Control)	7.43 ± 0.07^i^	50.95 ± 0.56^de^	53.49 ± 0.00^c^	3.34 ± 0.05^b^
WPP				
2.5	5.86 ± 0.11^k^	51.80 ± 0.36^d^	57.48 ± 2.93^b^	2.12 ± 0.05^d^
5	6.94 ± 0.05^j^	49.45 ± 1.89^e^	54.45 ± 0.09^c^	1.47 ± 0.12^e^
7.5	7.64 ± 0.14^hi^	46.61 ± 0.90^f^	53.46 ± 1.08^c^	1.43 ± 0.03^e^
10	8.94 ± 0.02^g^	45.51 ± 1.93^f^	52.38 ± 0.00^c^	1.29 ± 0.07^e^
15	10.68 ± 0.01^de^	41.39 ± 0.35^g^	48.68 ± 1.32^d^	1.18 ± 0.35^e^
WRP				
3.75	10.63 ± 0.05^e^	51.13 ± 1.09^de^	56.86 ± 2.32^b^	3.30 ± 0.08^b^
7.5	10.94 ± 0.34^d^	53.92 ± 1.62^bc^	60.00 ± 0.00^a^	3.22 ± 0.04^b^
11.25	11.42 ± 0.10^c^	55.55 ± 0.26^a^	60.21 ± 0.21^a^	3.18 ± 0.04^b^
15	13.09 ± 0.20^a^	56.16 ± 1.05^ab^	60.76 ± 0.77^a^	2.62 ± 0.53^c^
WSP				
5	7.73 ± 0.26^h^	50.67 ± 0.94^de^	56.98 ± 0.46^b^	3.17 ± 0.03^b^
10	9.44 ± 0.12^f^	52.59 ± 0.69^cd^	57.72 ± 1.47^b^	3.50 ± 0.05^b^
15	12.59 ± 0.21^b^	57.16 ± 0.04^a^	62.12 ± 1.34^a^	5.30 ± 0.60^a^

*Note:* ^a–k^There is no statistical difference between the data shown with the same letter in the same column (one‐way ANOVA; Duncan test; *p* ≤ 0.05).

Abbreviations: FP, Fresh pasta; OCT, Optimum Cooking Time; WPP, Watermelon peel powder; WRP, Watermelon rind powder; WSP, Watermelon seed powder.

The acceptable cooking loss limit for pasta is 8% (Bianchi et al. 2021; Long et al. 2024). According to Table 4, fresh pasta samples containing up to 10% watermelon peel and 5% watermelon seed comply with this limit. However, all pasta samples enriched with watermelon rind exceed this acceptable cooking loss value. The pasta sample with the highest cooking loss is the one containing 15% watermelon rind, which reached 11.65%, exceeding the acceptable limit of 8%, while samples with 2.5% WPP remained as low as 6.43%. In pasta enriched with different particle sizes of watermelon rind, it has been reported that samples produced with smaller rind particles exhibit lower cooking loss, while cooking loss increases as particle size increases (Long et al. 2024). This finding explains why finely ground watermelon peel results in lower cooking loss. The high cooking loss observed in pasta with 15% watermelon rind is attributed to excessive expansion during cooking (Table 2) and the compositional properties of watermelon rind. Similarly, pasta enriched with carob has shown increased cooking loss due to its high dietary fiber content (Zahorec et al. 2023).

In all pasta samples enriched with watermelon by‐products, cooking loss increased with higher incorporation levels. This increase may be due to the dilution of gluten networks. In pasta produced with watermelon seed, the high oil content of the seed may have delayed starch gelatinization, contributing to increased cooking loss.

In pasta samples enriched with watermelon rind and seed, the Weight Increase Index (WII) increased with higher incorporation levels, whereas this value decreased in fresh pasta samples enriched with watermelon peel. Finely ground watermelon peel disrupted the gluten network of the pasta, leading to structural disintegration and a subsequent decrease in the Weight Increase Index. According to Long et al. (2024), a decrease in particle size and an increase in dietary fiber content enhance water absorption, thereby increasing the Weight Increase Index in pasta. The results for pasta samples enriched with watermelon rind and seed in this study are consistent with these findings.

For the Volume Increase Index, a decrease was observed only in samples enriched with watermelon peel, as shown in Table 4. Despite the increase in cooking loss for pasta samples enriched with watermelon rind and seed, their volume increased, likely due to the high protein and carbohydrate content in these by‐products. The proteins and starches in rind and seed absorbed water during cooking, contributing to volume expansion.

An increase in protein content negatively affects cooking time and texture firmness (Moayedi et al. 2021; Acun and Gül 2023). Additionally, larger particles can enhance water diffusion, accelerating starch gelatinization and shortening cooking time (Alzuwaid et al. 2020). However, production methods and the pasta‐making process may have an inverse effect (Moayedi et al. 2021). Pasta enriched with watermelon seed, which contains larger particles than other watermelon by‐products, would be expected to have a shorter cooking time. However, the oil content in watermelon seed may have reduced heat transfer, leading to an extended cooking time.

Fresh pasta enriched with various plant‐based ingredients such as cinnamon, carob, and parsley has been reported to have cooking times ranging between 4.3 and 4.5 min. The lower cooking times observed in the pasta samples used in this study may be due to the structural disintegration of the pasta (Zahorec et al. 2023).

### 3.5 Total Phenolic Compound and Antioxidant Activity of FP

Watermelon by‐products, particularly the rind, are rich in phenolic compounds and exhibit significant antioxidant activity. These by‐products, which are often discarded, have potential for nutritional and functional applications due to their bioactive compounds. Watermelon by‐products, including the rind, are abundant in phenolics, flavonoids, and citrulline, which contribute to their antioxidant properties. These by‐products have potential applications in the development of functional foods, nutraceuticals, and pharmaceuticals due to their therapeutic properties, including antioxidant, anti‐inflammatory, and antibacterial effects (Zia et al. 2021).

The addition of watermelon rind to pasta significantly increases the product's phenolic content. Watermelon rind is rich in phenolic compounds with antioxidant properties. When watermelon rind or seed flour is incorporated into pasta, the phenolic content increases substantially, improving the nutritional profile of the pasta. As the incorporation level of watermelon by‐products increases, there is a corresponding rise in the total phenolic content of the pasta. The highest increase was observed in pasta fortified with watermelon rind. When compared at the lowest levels of incorporation, pasta containing 5% watermelon seed flour exhibited the lowest total phenolic content (3.90 mg GAE/g). In parallel with the increase in total phenolic content, the antioxidant activity also increased. The addition of watermelon by‐products to pasta enhanced antioxidant activity (measured as DPPH radical scavenging % inhibition) by 89.26%–628.86%, with pasta fortified with 15% watermelon rind showing the highest antioxidant activity (10.86%).

The cooking process significantly influences the amount and bioavailability of phenolic compounds in pasta. Generally, heat application during cooking can alter the structure of phenolic compounds in pasta, in some cases reducing their quantities while in others facilitating an increase and improving digestion (Padalino et al. 2019; Abbasi Parizad et al. 2020; Iván et al. 2023). The cooking method, duration, and temperature are critical factors affecting the retention or loss of phenolic compounds (Melini et al. 2020). In pasta enriched with watermelon by‐products, post‐cooking total phenolic content losses ranged from 1.17‐ to 11.11‐fold. The least loss was observed in pasta containing 15% watermelon seed flour, likely due to the high fat content of watermelon seeds. Since fat is a poor heat conductor, it may have contributed to the preservation of phenolic compounds. Similar to the phenolic compounds, the highest antioxidant activity (DPPH % inhibition) after cooking was observed in pasta with the highest incorporation levels of watermelon by‐products (Table 5).

**TABLE 5:** Total phenolic content and antioxidant activity of fresh pasta produced by adding watermelon by‐products.

Substitution levels of WPP, WRP and WSP (%)	Uncooked		Cooked	
	Total Phenolic Content (mg GAE/g)	Antioxidant Activity (%)	Total Phenolic Content (mg GAE/g)	Antioxidant Activity (%)
0 (Control)	1.85 ± 0.87^iA^	1.49 ± 0.44^jA^	0.31 ± 0.16^hB^	0.21 ± 0.00^hB^
WPP				
2.5	5.62 ± 0.54^ghA^	3.91 ± 0.40^ghA^	0.75 ± 0.00^ghB^	2.16 ± 0.15^fB^
5	9.57 ± 0.42^eA^	4.77 ± 0.50^efA^	1.78 ± 0.02^fgB^	3.03 ± 0.04^dB^
7.5	16.93 ± 0.46^cdA^	5.53 ± 0.28^cA^	2.79 ± 0.03^efB^	3.31 ± 0.04^cdB^
10	20.90 ± 1.84^bA^	7.08 ± 0.45^bA^	4.78 ± 0.72^dB^	4.21 ± 0.02^bB^
15	28.85 ± 2.00^aA^	10.86 ± 0.44^aA^	7.81 ± 0.86^cB^	4.93 ± 0.50^aB^
WRP				
3.75	4.22 ± 0.15^hA^	2.82 ± 0.29^iA^	0.38 ± 0.29^ghB^	0.35 ± 0.28^hB^
7.5	7.04 ± 0.23^fgA^	3.39 ± 0.38^hiA^	3.77 ± 1.31^deB^	1.02 ± 0.09^gB^
11.25	15.87 ± 1.09^dA^	3.60 ± 0.16^hA^	8.66 ± 0.99^bcB^	2.19 ± 0.09^fB^
15	18.99 ± 0.48^bcA^	5.17 ± 0.32^cdA^	10.14 ± 1.78^bB^	3.29 ± 0.11^cdB^
WSP				
5	3.90 ± 1.18^hiA^	4.03 ± 0.07^ghA^	2.41 ± 0.70^efB^	1.93 ± 0.01^fB^
10	8.15 ± 2.08^efA^	4.41 ± 0.32^fgA^	6.95 ± 0.00^cB^	2.57 ± 0.02^eB^
15	17.06 ± 1.97^cdA^	5.06 ± 0.00^cdeA^	13.95 ± 1.99^aB^	3.54 ± 0.00^cB^

*Note:* ^a–i^There is no statistical difference between the data shown with the same letter in the same column. ^AB^There is no statistical difference between applications shown with the same letter. (one‐way ANOVA; Duncan test; *p* ≤ 0.05).

Abbreviations: FP, Fresh pasta; WPP, Watermelon peel powder; WRP, Watermelon rind powder; WSP, Watermelon seed powder.

A study conducted by Long et al. (2022) reported that the addition of 25% watermelon rind to pasta production led to an 8.5‐fold increase in phenolic compound content and a 7.5‐fold increase in DPPH activity. Similarly, the incorporation of 15% watermelon rind resulted in approximately a 15.6‐fold increase in total phenolic content in raw pasta. Additionally, in another study by Long et al. (2024), it was demonstrated that the particle size of watermelon powders used in pasta production significantly affects the phenolic compound content of the final product. These findings highlight the substantial potential of watermelon rind incorporation for improving the nutritional value and antioxidant properties of pasta products.

### 3.6 Texture Profile Analysis of FP

Table 6 presents the TPA of cooked FP enriched with different rates of WPP, WRP, and WSP, detailing parameters such as firmness, springiness (also referred to as elasticity), cohesiveness, chewiness, and resilience.

**TABLE 6:** Texture profile analysis of cooked fresh pasta produced by adding watermelon by‐products.

Substitution levels of WPP, WRP and WSP (%)	Firmness (g)	Cohesiveness	Chewiness	Resilience
0 (Control)	378.66 ± 3.88^a^	0.84 ± 0.06^a^	300.51 ± 1.67^a^	1.91 ± 0.11^a^
WPP				
2.5	328.80 ± 25.96^bc^	0.77 ± 0.09^abc^	244.59 ± 5.02^b^	1.76 ± 0.22^ab^
5	304.63 ± 7.88^cd^	0.70 ± 0.02^cde^	195.53 ± 4.24^f^	1.54 ± 0.26^bc^
7.5	233.80 ± 22.64^e^	0.66 ± 0.02^e^	178.45 ± 7.75^h^	1.42 ± 0.10^cd^
10	168.85 ± 15.33^f^	0.58 ± 0.03^f^	87.14 ± 1.86^j^	1.06 ± 0.10^ef^
15	90.56 ± 33.06^g^	0.55 ± 0.00^f^	46.39 ± 0.81^l^	0.89 ± 0.05^f^
WRP				
3.75	353.72 ± 4.75^b^	0.78 ± 0.04^ab^	239.97 ± 2.61^bc^	1.71 ± 0.02^ab^
7.5	309.78 ± 11.62^cd^	0.74 ± 0.01^bcd^	233.51 ± 3.25^c^	1.71 ± 0.09^ab^
11.25	272.43 ± 42.58^d^	0.71 ± 0.00^bc^	224.64 ± 1.72^d^	1.60 ± 0.09^bc^
15	121.04 ± 24.28^g^	0.68 ± 0.01^de^	75.83 ± 1.87^k^	1.27 ± 0.07^de^
WSP				
5	274.28 ± 12.67^d^	0.72 ± 0.01^bcde^	171.75 ± 1.68^i^	1.42 ± 0.12^cd^
10	295.11 ± 4.92^cd^	0.71 ± 0.01^bcde^	187.96 ± 6.61^g^	1.57 ± 0.03^bc^
15	314.08 ± 13.74^c^	0.77 ± 0.08^ab^	205.64 ± 4.65^e^	1.63 ± 0.05^bc^

*Note:* ^a–l^There is no statistical difference between the data shown with the same letter in the same column (one‐way ANOVA; Duncan test; *p* ≤ 0.05).

Abbreviations: FP, Fresh pasta; WPP, Watermelon peel powder; WRP, Watermelon rind powder; WSP, Watermelon seed powder.

A significant reduction in firmness was seen when comparing the control pasta with cooked FP made with varying quantities of the WPP, WRP, and WSP substitutions. The proposed explanation is the formation of a weaker gluten network by the addition of WBP fiber. These dietary fibers can disrupt the gluten networks, leading to their dilution and subsequent weakening, which results in pasta losing its firmness (Namir et al. 2022). The results are in line with those exposed by Bchir et al. (2022), showing that the increase in pear, date, and apple by‐products supplementation (0, 2.5, 5, 7, and 10 g/100 g) caused a decrease in firmness (from 12.50 N to 7.24 N). Moreover, many authors reported lower firmness with pasta made with different by‐products, namely grape peels by‐product (Ungureanu‐Iuga and Mironeasa 2021), potato peel by‐product (Namir et al. 2022), raspberry pomace powder by‐product of raspberry juice (De Santis et al. 2022). In contrast, a firmness‐increasing impact of trub, a beer industry by‐product, was measured in durum wheat fresh pasta (Lomuscio et al. 2022). The textural hardness of pasta containing different dietary fibers can vary significantly due to several factors, including the type of fiber, its particle size, the intrinsic amount of incorporation, the interaction with the pasta matrix, and the pasta production method. However, the firmness of FP decreased significantly as more WPP and WRP were added, while the samples that contained WSP exhibited the opposite trend. Firmness increased from 12.13 N in the control to 14.82 N in pasta containing 15% WSP, whereas it decreased to 7.45 N in pasta containing 15% WPP. In other words, when the concentration of WSP raised, the firmness of FP likewise increased. This could arise from the greater particle size of WSP in comparison to WRP and WPP. In our previous research (Acun et al. 2025), we determined that the WSP had the highest particle size among the three samples, with the bulk (78.51%) exhibiting a particle size above 446 μm. Finer particle sizes of WPP and WRP than WSP can soften the pasta texture. Additionally, the results of Long et al. (2024) indicate that reducing the particle size of watermelon rind powder decreased pasta hardness by 13%. Conversely, larger particles may create a stiffer structure, as seen with wheat bran and mucilaginous seeds, which resulted in a denser pasta matrix (Naji‐Tabasi et al. 2021).

The cohesiveness of FP showed a decreasing trend with the addition of WBP. The cohesiveness of the FP decreased as the WPP ratio increased. When WPP and WRP were used at low ratios, they did not have a significant effect on the cohesiveness of FP, but when the substitution rate was increased above 5%, they started to have a decreasing effect on this value. This indicates that as the dietary fiber content increases, the gluten network structure of the FP deteriorates and they have a weaker texture and less force is required to hold the components together. WSP showed no statistical difference between the different supplementation levels, while there was no significant difference with the control at the 15% substitution level. This is due to the high fat content of WSP than WPP and WRP (Acun et al. 2025). This fat may have had a cohesive effect between the components.

Chewiness was significantly reduced in all WBP‐added FP samples compared to the control. In WPP‐ and WRP‐substituted FP, chewiness values decreased as the substitution rate increased. However, the WSP‐substituted samples exhibited a different trend: chewiness increased with increasing WSP levels. While FP samples containing 15% WPP and WRP had the lowest chewiness values among all formulations, the chewiness of FP with 15% WSP was markedly higher. This increase may be attributed to the higher fat content of WSP compared to WPP and WRP.

While the resilience of the control was determined to be the highest, there was no significant difference between the samples in which WPP and WRP were substituted at low levels. Resilience decreased steadily when the substitution rate was increased from 2.5% in WPP to 7.5% in WRP. WSP also had a different effect on resilience compared to the other two, and this rate increased as the substitution rate increased. However, it still showed lower values than the control.

### 3.7 Sensory Evaluation

**FIGURE 5:**
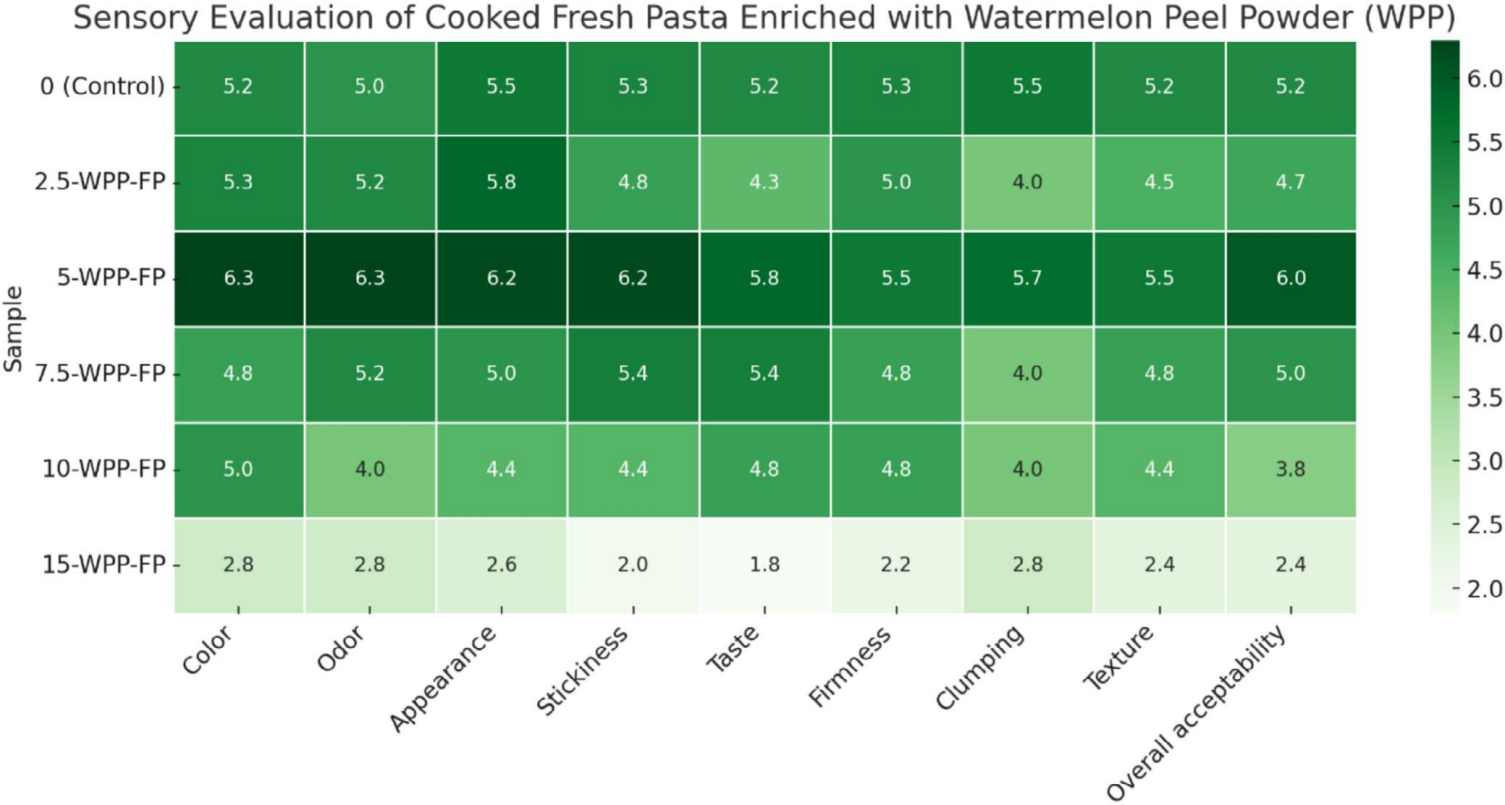
Sensory evaluation results of cooked fresh pasta supplemented with different levels of (0%, 2.5%, 5%, 7.5%, 10%, 15%) watermelon peel powder, FP, Fresh pasta; WPP, Watermelon peel powder.

**FIGURE 6:**
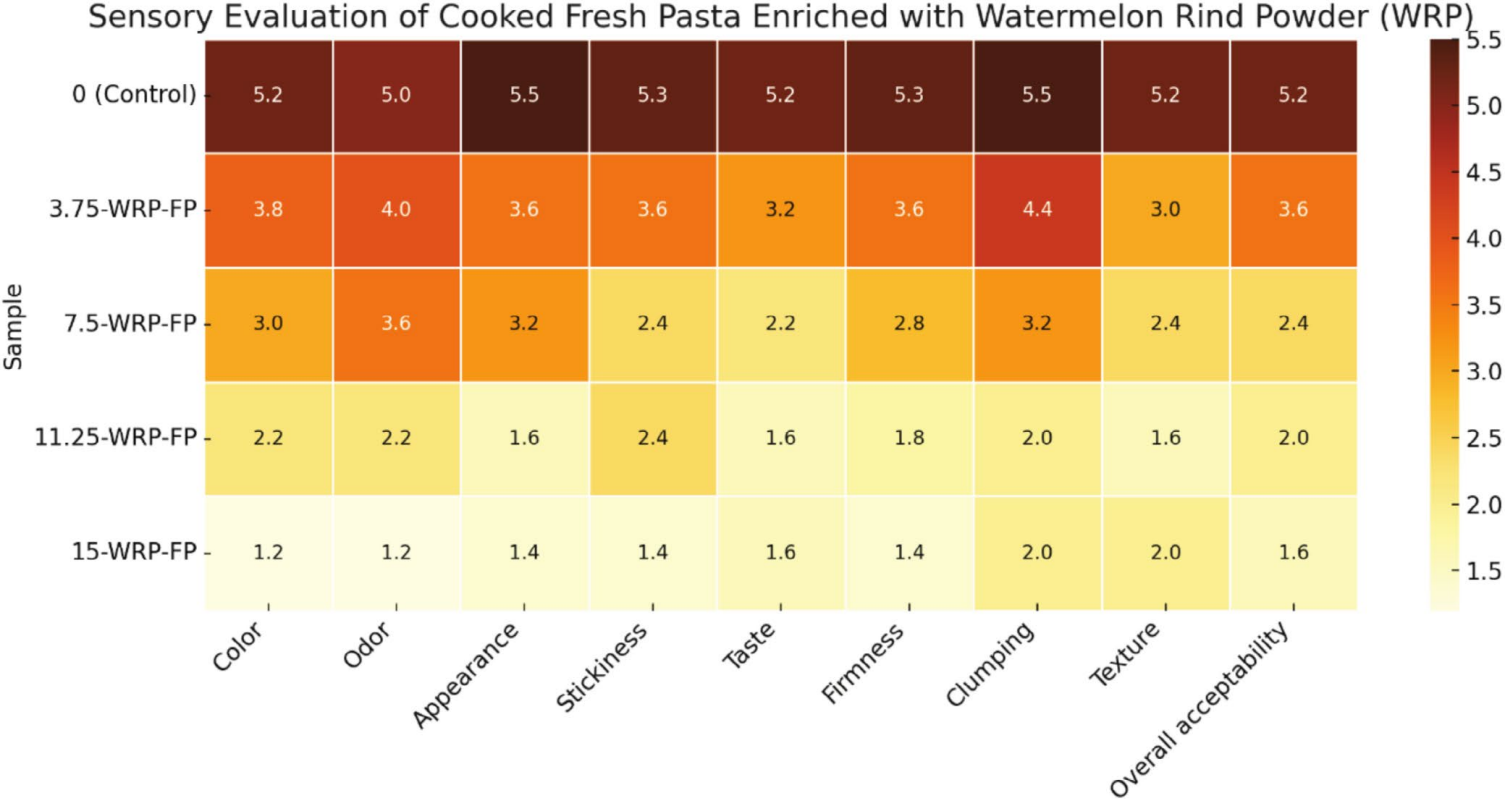
Sensory evaluation results of cooked fresh pasta supplemented with different levels of (0%, 3.75%, 5%, 7.5%, 11.25%, 15%) watermelon rind powder. FP, Fresh pasta; WPP, Watermelon rind powder.

**FIGURE 7:**
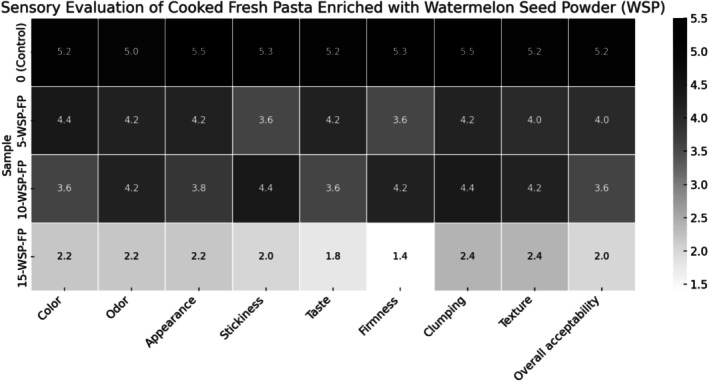
Sensory evaluation results of cooked fresh pasta supplemented with different levels of (0%, 5%, 10%, 15%) watermelon seed powder. FP. Fresh pasta; WSP, Watermelon seed powder.

Figures 5, 6, 7 present the sensory evaluation results of WPP, WRP, and WSP substituted FP, respectively. The FP sample with 5% WPP received the highest ratings for all sensory attributes, as well as for overall acceptability and affordability, when evaluated independently. This sample was even more appreciated than the control. The 2.5‐WPP‐FP, 7.5‐WPP‐FP, and 10‐WPP‐FP samples exhibited comparable scores, however the 15% WPP addition received the lowest score. This is because, as Figure 5 illustrates that when the additive rate is raised to very high levels, such as 15%, the FP fails to preserve its structural integrity and subsequently disintegrates. The fact that pasta is not accepted primarily in terms of appearance has negatively affected all its other properties. Consequently, the overall quality and consumer acceptance of the final product diminished, suggesting that excessive amounts of the WPP can compromise not only the visual appeal but also the textural and flavor characteristics desirable in FP. This highlights the need for a balanced formulation to achieve optimal results without sacrificing essential attributes. Accordingly, our findings are in line with the suggestion of Drabińska et al. (2022), who noted that the pasta fortified with up to 5% broccoli leaf powder was rated very high, indicating that it remains acceptable to consumers in terms of taste, odor, appearance, and texture.

Significant statistical differences were observed between the WRP‐added FP and the control sample for individual sensory attributes, including appearance, color, odor, texture, and taste. Following the evaluation, 3.75‐WRP‐FP emerged as the most favored FP in terms of all sensory attributes and general acceptability (Figure 5). Despite the increase in the WRP replacement rate, all sensory scores of the FP decreased proportionately. Likewise, Long et al. (2024) indicated that incorporating watermelon rind flour into pasta led to a decrease in the total sensory evaluation score. Pandit et al. (2020) conducted a sensory evaluation to assess the acceptability of spaghetti made from watermelon rind and agar, and they concluded that watermelon rind spaghetti, both sweet and savory, is a novel and acceptable product. This indicates the potential for using fruit waste in creating low‐energy, health‐beneficial food products that are appealing to consumers. Although the incorporation of watermelon rind flour into pasta enhances its nutritional profile, it presents difficulties regarding sensory quality, especially at higher supplementation levels.

Figure 6 shows that the sensory scores of FP containing WSP were closest to the control at the 5% and 10% addition levels, particularly for texture and overall appearance. However, the sensory attributes diminished when the substitution level rose to 15%. This could be attributed to the larger particle size of WSP, which causes a coarser texture in the pasta at higher incorporation levels. In pasta with 15% WSP substitution, black spots appeared, which negatively influenced consumer preferences due to a general preference for uniformly colored pasta.

**FIGURE 8:**
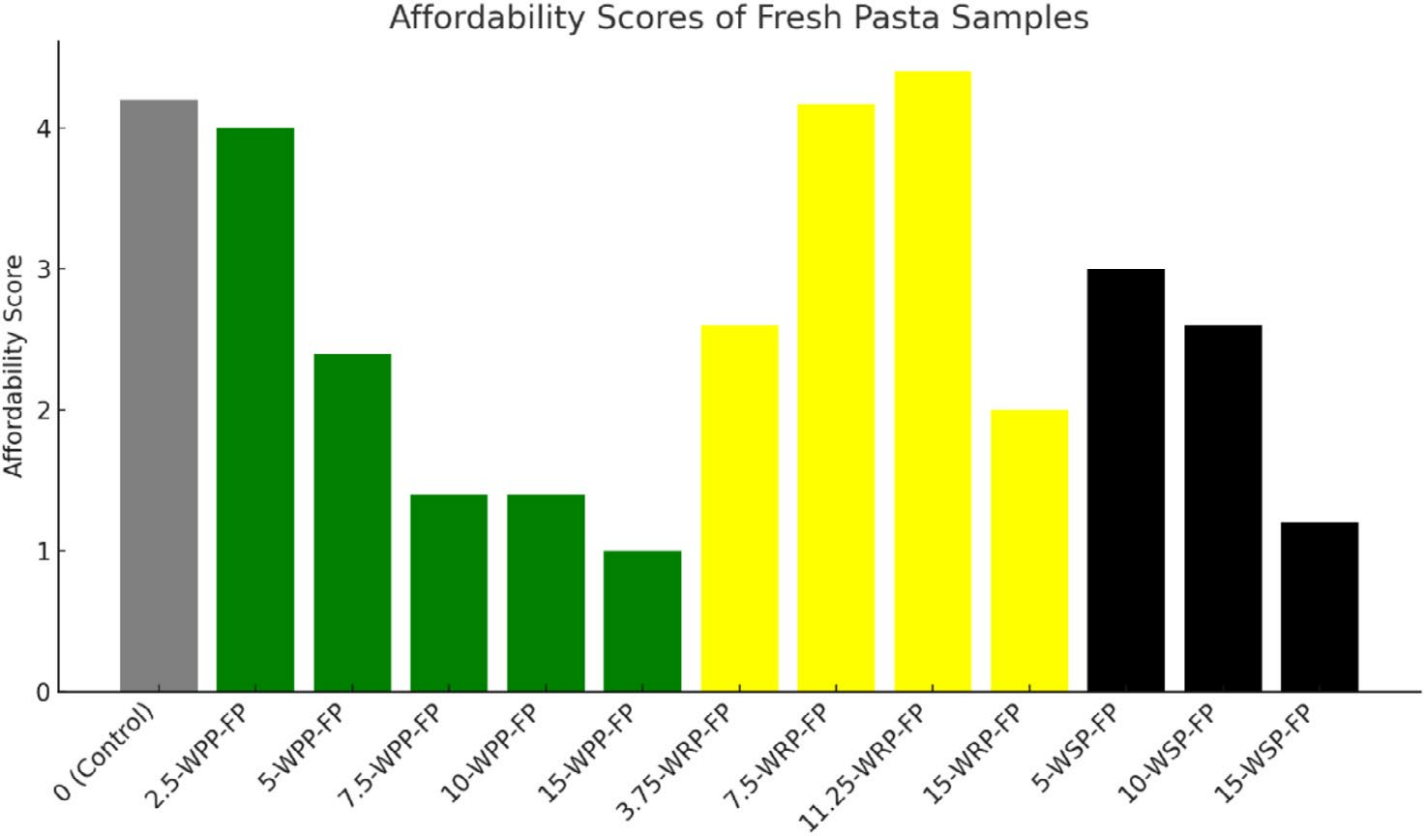
Affordability scores of cooked fresh pasta (FP) enriched with varying levels of watermelon by‐products. WSP, Watermelon seed powder (0%, 5%, 10%, 15%); WRP, Watermelon rind powder (0%, 3.75%, 7.5%, 11.25%, 15%); WPP, Watermelon peel powder (0%, 2.5%, 5%, 7.5%, 10%, 15%). Affordability was evaluated by panelists independently of sensory attributes, based on perceived value for money.

Our investigation established that the sensory attributes of pasta—namely appearance, color, odor, texture, taste, and overall acceptability—can be markedly affected by the use of diverse fruit by‐products and alternative flours. Bchir et al. (2022) reported that pasta enriched with 2.5% by‐products (apple, pear, and date by‐products) received the highest overall acceptability score, while acceptability decreased significantly as by‐product content increased from 5% to 10%, indicating that higher by‐product ratios were less liked by consumers.

In addition to sensory evaluation, consumer purchase intention was also assessed separately. The 3.75‐WRP‐FP sample showed the highest level of affordability perception among panelists. Upon reviewing all the FPs collectively, it was determined that the optimal incorporation levels of WBPs were 5% for WPP, 3.5% for WRP, and 10% for WSP. These levels may be utilized to produce FPs with improved technological functionality and acceptable sensory characteristics. Although innovation in pasta formulations is crucial for enhancing nutritional value, sensory quality must be carefully considered to avoid consumer rejection.

Figure 8 shows the affordability scores of fresh pasta samples enriched with various concentrations of watermelon by‐products. The control sample received a moderately high affordability score of 4.2, serving as the reference point for comparative analysis. Among all enriched formulations, the sample containing 2.5% WPP exhibited a similar affordability score (4.0), indicating consumer acceptability at lower incorporation levels of watermelon peel.

However, as the WPP level increased, a clear declining trend in affordability was observed, with the 15% WPP formulation receiving the lowest score (1.0) among all groups. This suggests that high levels of WPP may have negatively influenced visual, texture, or perceived quality attributes relative to cost.

In contrast, pasta samples enriched with WRP displayed an increasing trend in affordability up to 11.25% inclusion, which received the highest score (4.4) overall. This implies a favorable balance between perceived value and product characteristics at mid‐level WRP concentrations. Nonetheless, further increases (e.g., 15%) led to a decline, potentially due to over‐fortification effects.

Formulations containing WSP achieved intermediate affordability ratings. The 5% and 10% WSP samples were generally perceived as comparable to the control, while the 15% WSP variant was rated significantly lower, likely reflecting negative perceptions at higher inclusion levels.

These findings suggest that affordability perception is dose‐dependent and varies across by‐product type. While moderate incorporation of WRP may enhance perceived value, higher levels of WPP and WSP may reduce consumer acceptance from an economic standpoint. Therefore, optimization of by‐product inclusion levels is essential to ensure a balance between nutritional enhancement, sensory appeal, and cost‐related acceptability in novel pasta formulations.

## 4 Conclusion

This study has demonstrated the effects of incorporating watermelon by‐products into fresh pasta on its nutritional composition, cooking quality, and color characteristics. The findings indicate that different watermelon by‐products enrich the composition of pasta, thereby enhancing its nutritional value. Specifically, the addition of 15% WPP, due to its high dietary fiber content, disrupted the gluten network integrity, leading to fragmentation and a decrease in hardness. The addition of 15% WSP, with its high fat content, extended the cooking time while increasing the hardness of the pasta. The addition of 15% WRP resulted in an increase in cooking loss, which negatively affected the structural integrity of the pasta.

Color analysis revealed that the incorporation of by‐products caused significant variations in pasta color. The addition of WRP gave the pasta a more yellowish hue, while WBP imparted a greenish tone, and WSP resulted in a darker color. Additionally, the total phenolic content and antioxidant activity of fresh pasta increased in proportion to the amount of by‐product added.

From a technological perspective, it was determined that incorporating watermelon by‐products at a 10% level positively influenced the quality characteristics of fresh pasta. In this context, the use of watermelon by‐products may offer a sustainable and functional enrichment strategy for fresh pasta, particularly by enhancing its dietary fiber content and antioxidant potential. Future studies should focus on evaluating the bioavailability of minerals and phenolic compounds during digestion, as well as assessing consumer acceptance. This would support sustainable strategies for reducing food waste while creating new opportunities for the production of functional foods.

## Author Contributions

**Sultan Acun:** conceptualization (equal), formal analysis (equal), funding acquisition (equal), investigation (equal), methodology (equal), project administration (equal), writing – original draft (equal), writing – review and editing (equal). **Hülya Gül:** conceptualization (equal), methodology (equal), supervision (equal), writing – original draft (equal), writing – review and editing (equal). **Fadime Seyrekoğlu:** investigation (supporting).

## Ethics Statement

The authors have nothing to report.

## Consent

The authors have nothing to report.

## Conflicts of Interest

The authors declare no conflicts of interest.

## Data Availability

The data supporting the findings of this study are available from the corresponding author upon reasonable request.
